# ‘Silence is gratitude’: social support and female empowerment to improve health and well-being in the Pakistani community, UK

**DOI:** 10.1186/s12913-025-13220-8

**Published:** 2025-08-12

**Authors:** Farina Kokab, Sheila Greenfield, Antje Lindenmeyer, Manbinder Sidhu, Paramjit Gill

**Affiliations:** 1 Department of Social Work and Social Care, The University of Birmingham, Edgbaston, Birmingham, England, B15 2TT UK; 2 Institute of Applied Health Research, The University of Birmingham, Edgbaston, Birmingham, England, B15 2TT UK; 3 School of Nursing, The University of Birmingham, Edgbaston, Birmingham, England, B15 2TT UK; 4 The Health Services Management Centre, University of Birmingham, Edgbaston, Birmingham, England, B15 2TT UK; 5 Warwick Medical School, Warwick, Coventry, CV4 7AL UK

**Keywords:** Qualitative, Pakistani, Women, Social capital, Identity

## Abstract

**Background:**

South Asian migrants living in high income countries have an increased risk for developing cardiovascular disease (CVD), but there is limited research exploring the underlying socio-cultural causes and specifically as they impact women.

**Methods:**

Social capital was used as an interpretive lens to analyse of in-depth qualitative interviews across three generations of at-risk migrant Pakistani women living in the West Midlands, UK. Perceptions of trust, cultural norms and social networks were the primary areas of exploration.

**Results:**

Findings highlighted the importance of educational and occupational opportunities to seek healthcare related support outside of the Pakistani community. Appearance played a key role as there were varying ideals for younger or older women depending on how participants perceived their role in society. Women were often navigating pluralistic identities depending on whether they were at home or outside of their community setting. There were limited social spaces for women to seek support or information for their health as local sites, such as gyms, felt were prioritised for men.

**Conclusions:**

There were age-based and generational differences in how women in the Pakistani community perceived the role of their social networks and community spaces in helping them to develop healthier lifestyle behaviours. The pursuit of a healthier lifestyle varied across the diverse migrant community, and efforts are needed to promote wider integration between communities and locations with the potential needed to improve to health.

## Background

Inequalities in cardiovascular disease (CVD) can reflect social and racial discrimination present in wider society, where Black and South Asian communities (i.e. those originating from India, Pakistan, Bangladesh) experience varying degrees of social disadvantage that puts them at risk [7, 15, 38]. Research in the UK shows a higher prevalence and mortality with CVD in South Asian communities compared with their White British counterparts [38]. Women migrating from India, Pakistan and Bangladesh face health risks within a broader socio-cultural context that may influence their health-seeking behaviour, for example where the risks of CVD can be increased through limited opportunities to engage in physical activity [34]. In the UK, the 10-year National Health Service (NHS) Long Term plan, first published in 2019, focused on the need to develop more significant support for people from minority ethnic backgrounds to address specific health needs through lifestyle services. However, challenges remain about how best to engage BAME communities and raise awareness on how individuals from diverse backgrounds can make healthier choices local to where they live [25].

The South Asian diaspora is one of the largest migrant groups in the UK [31]. There is a growing cohort of second and third generation descendants trying to accommodate traditional identities with Western influences [10].

When viewed at the neighbourhood level, minority ethnic groups, especially Pakistani and Bangladeshi, experience higher levels of deprivation compared to their White counterparts even within the same geographical area [27]. Network-based resources can be invaluable to recent migrants who can benefit from the security of established social networks, support to find employment and housing, as well as accessing healthcare services that are not readily available elsewhere [19]. The protection afforded in migrant networks is known as the ‘buffering’ effect where individuals are better shielded from perceived racism but encounter a potential drawback of living in poorer conditions with greater exposure to cheaper fast-food outlets compared to markets providing healthier alternatives at low cost [35]. Evidence suggests minority-ethnic men and women could benefit from socio-political spaces and networking opportunities that positively shape and influence their health behaviours [16].

This work addresses the unique intersection between cultural identity and health for women who are balancing their South Asian and predominantly Muslim identities with Western norms while facing gender-related challenges [25]. Employed Muslim women, for instance, must consider religious guidelines on gender interactions when choosing a profession, a concern less common in other communities [41]. They must also navigate mixed-gender spaces shaped by patriarchal norms that limit their participation in decision-making, contradicting Islamic principles that emphasize modesty and women’s protection in public spaces [28].

Social capital helps explain how social and cultural beliefs influence health behaviours in migrant groups. It refers to resources gained through social networks based on trust and reciprocity [19, 35]. Minority-ethnic groups are often assumed to have support systems for managing health, but limited social capital can exacerbate health inequalities among Pakistani women by restricting healthcare access, increasing isolation, and worsening economic and mental health challenges [20, 24].

Bonding social capital provides emotional support within the Pakistani community, reinforcing traditional norms, while bridging social capital expands networks, offering new resources and alternative health choices. However, cultural and language barriers, economic disadvantages, and distrust in healthcare providers worsen these disparities. For instance, Pakistani women in the UK may struggle to balance medical advice with traditional community wisdom, especially during pregnancy [8, 18, 29]. Understanding cultural context is crucial to promoting healthier lifestyles whilst recognising their autonomy over health decisions.

This paper aims to examine how social capital, through bonding and bridging networks, can influence the health behaviours and self-care practices of Pakistani women, particularly in managing chronic disease risk factors, while navigating cultural and societal expectations within their communities.

## Method

### Aim

The present research explores Pakistani women’s views on health behaviours and lifestyle choices related to CVD prevention, including (but not limited to) diet and exercise, as well as social support for achieving these behaviours within and outside of their local community.

### Design

A community-based interpretive qualitative study based on in-depth, face-to-face interviews [25]. Social capital theory was used as an interpretive lens to understand how social networks, trust and cultural norms may shape Pakistani women’s access to health information and support [19, 25].

### Setting

Recruitment undertaken October 2013-February 2014 across the West Midlands region—predominantly in Birmingham, UK. Birmingham is the UK’s second-largest populated city and home to a large Pakistani community comprised of 195,000 individuals, with one of the highest proportions of South Asian residents in the country [32]. Census data shows that Birmingham remains a city with a significant (31%) South Asian population, with Pakistani individuals being the largest non-white ethnic group (13.5%) [32].

### Sampling, access, and recruitment

To successfully recruit a diverse sample of Pakistani women an inclusive approach was taken whereby issues with literacy, language, cultural advocacy for participating in the research (trust and sense of belonging), knowledge of research, obtaining consent, and travel to research facilities were considered. A researcher from a similar ethnic background (FK) encouraged participation and shared an understanding of socio-cultural norms and provided the opportunity to take part in interviews in either Urdu or English.

Purposive sampling was used to recruit Pakistani participants from trusted community sites, such as businesses (e.g. hair salons) in high ethnic-density areas. Selection criteria included age, gender, and community involvement to ensure diversity. Business owners in Birmingham acted as ‘gatekeepers’ and ‘research advocates’ [25, 26], promoting recruitment through community connections. Additional methods included word-of-mouth, third-sector organisations, social media (i.e., Facebook), and snowballing. Recruitment information was shared, oral ads, posters, and volunteering services email lists.

### Developing the topic guide

The topic guide was piloted with four voluntary participants from the Pakistani community to test its ability to facilitate conversation in English and Urdu. Consequently, the guide was adapted to avoid dichotomous responses in Urdu as some questions became close-ended when translated. The piloted topic guide was designed to explore the influence of social networks, trust, and cultural norms (social capital) in creating and maintaining health behaviours related to CVD prevention with changes to better suit data collection [25]. Before the interview, participants were asked to complete a Convoy model diagram [3, 23] by mapping their social networks using concentric circles, with the closest relationship in the centre.

### Data collection

Participants interested in the research chose their interview location, including their home, workplace, recruitment site (e.g. hair salon), or the University. Most interviews (*n* = 20) were in English, with two interviews in predominantly Punjabi and Urdu. Participants often used Punjabi or Urdu words or phrases in their interview to better articulate their experiences during interviews, even when interviews were conducted in English.

### Ethics

Ethical approval was given by the University of Birmingham Science, Technology, Engineering, and Mathematics Ethical Review Committee application number: ERN_13-0450. All participants were provided with a verbal study explanation and an information sheet before giving written consent to take part in the research; they were informed of their right to withdraw. Data were stored in accordance with University of Birmingham ethical guidelines.

### Analysis

Interviews were audio-recorded, transcribed verbatim, anonymised, and translated where needed. Transcription and coding occurred simultaneously in an iterative process. NVivo 11 was used for data management. A South Asian researcher at the University verified transcripts for accuracy.

The Framework method [13] guided analysis through five stages: familiarisation, thematic framework identification, indexing, charting, and interpretation. Data was organised into five topic areas: competition for resources, socio-economic success, Pakistani identity, health-seeking behaviour, and gender inequalities organised as moving from community, through identity, to personal levels of reflection (see Table 1).

**Table 1:** Development of the thematic narrative (communal to personal perspective) [25]

Community	Being Pakistani	Personal factors
Competition for resources • Position and power • Public appearances • Health Vs. Financial prosperity • Trustworthy people	• Microcosm of religion and ethnicity • Roles of community members • Identity	Seeking health related help and information • Access to health • Institutions
Socio-economic success • Socio-economic status gradient • Traditional culture and socio-economic status • Exposure through education		Gender inequalities • Female appearance: education and liberty • Societal pressure on men

## Findings/results

### Participants

Participant characteristics are outlined in Tables 2 and 3.

**Table 2:** Characteristics of female participants

Generation	Age 18–29	Age 30–49	Age 50 and over	IMD	Education	Occupation	Marital status
First (*n* = 13)	4	8	1	1: (*n* = 9) 4: (*n* = 1) 5: (*n* = 2) 7: (*n* = 1)	University: 5 College: 2 Secondary school: 6	• Accountant • Educator • Beautician • High-street store manager • Cleaner (*n* = 2) • Carer • School dinner lady • Housewife (*n* = 4) • Student	Married: 9 Single: 4
Second (*n* = 7)	5	2	0	1: (*n* = 3) 2: (*n* = 1) 3: (*n* = 1) 5: (*n* = 2)	University: 6 Secondary school: 1	• Nurse • Product development • Housewife (n = 2) • Student (n = 3)	Married: 2 Single: 5
Third (*n* = 2)	2	0	0	1: (*n* = 2)	University: 2	• Teacher • Student	Single: 2

Index of Multiple Deprivation (IMD) level of 1 = least and 5 = most deprivation [12]

First-generation (Born in subcontinent migrated directly/indirectly to the UK)

Second generation (Born in the UK or received formal education from the age of 5)

Third generation (Born in the UK and at least one parent from the second-generation)

**Table 3:** Outline of the different areas in the West Midlands

Location in Birmingham (constituency, ward and area)	Description	Population total	Pakistani population
Area A	Centre of industrial revolution in the Black Country	312,952	10,399
Area B, G, J, and K	Multi-racial residential area with a diverse range of shops, contemporary restaurants and cafes	115,904	37,653
Area C	Middle-class residential area, Southwest of the city-centre, leafy parks and cricket ground	96,568	2844
Area D, I and L	One of the main centres for the Pakistani community but trends for moving outwards	126,693	19,484
Area E, F and H	Predominantly Pakistani (Mirpuri inhabitants) characterised by take-away restaurants and ethnic-specific shops	121,678	46,042
Area M	Majority non-White, close to the city-centre, occupied largely by West Indian and South Asian Immigrants	107,090	12,902

Iqbal [21], Sandwell Trends [37], Birmingham City Council [5], Dudley Metropolitan Borough Council [11], Kokab et al. [25]

We analysed the views of 22 women who were either first, second, and third-generation migrants, with a range of educational qualifications, and diverse occupational backgrounds (see Table 1), to better understand women’s perceptions of social support when accessing healthcare from socio-economically disadvantaged areas across the West Midlands, UK. Occupational opportunities included financial autonomy, access to broader social networks, and the ability to engage with non-community sources of health support. Occupational opportunities afforded both financial independence and exposure to alternative sources of health information and support outside of the Pakistani community. Majority of the women (*n* = 21,) were aged under 50 years.

### Themes

The encompassing themes include 1) how women’s education and employment are the building blocks for empowerment; 2) the importance of physical appearance as a signifier for social status, 3) female empowerment as facilitated by social networks, and 4) the force and the influence of patriarchy on health-seeking behaviour.

#### Education and employment as the building block for empowerment

More than half of the women in this study had higher educational qualifications (*n* = 13), almost a third (*n* = 7) had a professional career, and half (*n* = 11) paid employment across various industries. Education and work led to lifestyle changes, especially for younger women in academic or professional settings. These opportunities expanded social networks and provided access to health and well-being information beyond their immediate community.

‘My friends never said anything, but my colleagues they told me and gave me good support that you can do this (lose weight)’

(30–49 years, first-generation, housewife (formerly employed), IMD 1, area E)

Yet, despite such support, women struggled to manage these lifestyle changes alongside upholding their community’s cultural and religious values. Healthier lifestyle choices, which require motivation and determination with respect to food and exercise behaviours, were traditionally viewed as masculine concerns.

“it’s like, ‘what you playing sports for? You should be concentrating on doing this, become a lawyer, become a doctor, become…’…all the stereotypical views, like, become this, become that…like my brothers, they play football. Even my little brother’s ten years old, he’s part of football club.

(18–29 years, third-generation, student, IMD 5, area M)

For some women, achieving in a high paying profession and contributing financially became another family obligation which was added to caring responsibilities which left little time for physical pursuits. They highlighted the greater demands placed on Pakistani women compared to men:

‘Men want their women to go out and earn and then what they want them to do is pay half of their mortgages and bills and everything and you see some marriages- what is it? You see that in people, ‘Oh, I’ve got to do the…’ the women go, they expect to work, keep their living, look after their children, but what does the man do? He just goes out, does his work, comes home, puts up his feet’.

(30–49 years, first-generation, professional, IMD 7, area C)

#### Contradictory cultural influences on physical appearance

Participants also emphasised the importance of physical appearance in their community, particularly in securing social approval for marriage and building support networks. Young, unmarried women were expected to demonstrate cultural customs and maintain a specific appearance. In contrast, Pakistani men were expected to uphold grooming and fitness in line with traditional or religious norms.

### Signifier for social status

There were challenges associated with staying slim for women who had little control over food they ate and when exercise is difficult. The following participant describes the discourse around young Pakistani women’s self-presentation:

“being Pakistani and being part of that culture they have this set image of this pretty, good-looking perfect girl who has to have that height, who has to have that colour (fairness), that attitude and that size […] she was highlighting the fact that how pretty she is, she’s got straight brown hair, she is really fair, she has blue eyes and weight 47 kilos”

(18–29 years, first-generation, professional, IMD 1, area E)

Generationally, mothers played an important monitoring role over their daughters’ physical appearance according to participant accounts. Weight gain for a young single woman could have negative social consequences, and mothers tried to monitor their daughters’ appearance in the context of community hearsay:

“And especially about weight. If I’ve put on weight, she’s like ‘what’s that?’ -laughs- and I’m like ‘ooh, it just happens’ and she’s like, ‘no it doesn’t happen. Eat less’. She’ll always tell me whether I’ve put on weight, or not or whether I’ve just lost too much weight and I need to eat more.

(18–29 years, first-generation, student, IMD 1, area E)

### Difficulty in achieving the right physical appearance

Yet, participants felt community members would be critical of their efforts to maintain their physical appearance given the cultural and religious restrictions imposed upon them by their culture and faith:

“it’s the whole concept of covering whilst you’re running and she (mother) said ‘if I want to run, I’ll have to go to a place where no one is going to see’ […] I would never say to a Muslim girl its Islamic to run ‘cos when I used to run I used to cover (wear hijab), I would wear a cap because of the sweat and stuff but it’s hard for me to say to a girl that wears a hijab that go running, because it’s not, I can’t take that sin off you [for exposing yourself whilst running]”

(18–29 years, second-generation, professional, IMD 1, area F)

Given such scrutiny, participants expressed a desire to keep their personal lives private within their community. The stigma attached to improving one’s personal health through lifestyle changes considered culturally inappropriate was too great a risk for some participants to discuss publicly and many chose not to disclose information such as attending mixed gender gyms which was viewed akin to undesirable traits like smoking tobacco (a cultural taboo that is greater for women compared to men in the Pakistani community).

“I wouldn’t feel comfortable- like at home, because it was, like I was saying, I wouldn’t trust telling them that I go to the gym or that I smoke because of all the other connotations around it.”

(18–29 year old, professional, second-generation, IMD 1, area D)

Participants also kept their lifestyle choices private to avoid resistance or minimise conflict at home, especially when unmarried. Pakistani women, as well as other South Asian women, in their majority remain in their familial homes until they are married, where having an independent lifestyle (i.e. making decisions without elders’ approval and focusing on individual pursuits) was seen as fractious to familial dynamics [42]. For example, participants felt that making their own food choices would be seen as undermining the authority of their parents:

“I feel like it would be offensive…silence is gratitude…to be like, ‘okay, you don’t want the food’. Humbly take my portions of it or whatever. Again, it would be offensive to not eat what they buy, because then it’s like, ‘well, what’s wrong with what I bought you?’”

(18–29 years, professional, second-generation, IMD 1, area D)

Younger participants struggled to adopt healthier lifestyles due to a lack of social support and affirmation. They often felt insecure about their choices and hesitated to share experiences unless peers held similar beliefs. This created a paradox- wanting to discuss lifestyle changes but fearing judgement from a community with varying traditional values.

Some of the older or first-generation participants were concerned by women being superficially well presented in public, whilst ignoring their health and well-being. This is an example of the judgement that younger participants are referring to when trying to make independent choices (e.g. around food).

“The girls are balloon-y. Young, pretty, good skin, and lots of makeup because they’re really got into grooming, whereas we weren’t into grooming ‘cos we didn’t have the products or the money 30 years ago, or the know-how. These girls are very groomed, threaded eyebrows, lovely lashes, lovely grooming, lots of makeup, not necessarily good skin, sometimes really bad skin. A lot of them’ve got (overweight/obese), because it’s bad diet I think as well”.

(50 + years, first-generation, professional, IMD 7, area C)

Although physical appearance was often discussed, it was in relation to social acceptability and community perception rather than medical or health-orientated goals. The relationship between notions of physical appearance and health was contextualised around beauty and attractiveness: a trait valued by members of the Pakistani community acting as a signifier of status and acceptance. Yet, participants felt uneasy about the experience of being scrutinised and the wider obligation to appear socially and physically acceptable from the lens of *their* community. Those who were considered socially acceptable had greater access to resources and more personal freedom, including choices regarding partners for marriage. This could influence their health, as women were often seen engaging in exercise or dieting not to prevent cardiovascular disease (CVD), but to improve their chances of securing a desirable marriage (both partner and family)- an effort that was sometimes encouraged and at other times criticised.

#### Female empowerment facilitated by social networks

Participants recognised the importance of prioritising their health and challenging well-established inhibiting values regarding female health and well-being, gender stereotypes, or social networks. The following quote illustrates the prohibitions on men and women exercising together in public spaces, which is more acceptable for middle-aged members of the community.

“I’ve seen in the park-I see middle-aged men and women running together and I think it’s so adorable. These Pakistani women in their *shalwar kameez* but trainers [brand]gently jogging along”.

(18–29 years, second-generation, professional, IMD 1, area D)

Narrow social groups also spelt competition amongst community members with little space for participants to empower one another or use available social or economic resources. When comprised of similar type of people, social networks may only provide so many opportunities. Many participants expressed a desire to develop broader social groups to learn more about improving their well-being; but such people were seen as trailblazers rather than the norm.

“Everyone no matter how good friends or family, doesn’t matter how good, at some point you have to do your individual things, and everyone has to move away from there, otherwise you will always be caught up in that circle.”

(30–49 years, first-generation, housewife, IMD 1, area E)

Second -generation participants’ apparent empowerment is evidenced by social behaviours. Participants who perceived themselves as such trailblazers wanted to empower other women in the Pakistani community; thus, challenging social norms, so future younger women could make lifestyle choices without resistance from members of their own community. These trailblazers use their social networks/groups to facilitate this:

“I do feel like I’m fighting-I feel like I’ve always had to fight anyway, to have a sort of a social life because of what culture dictates, or my family’s sub-micro-culture dictates, but I feel like I’m having to fight it even more now and have to fight for myself in that way”.

(18–29 years, second-generation, professional, IMD 1, area D)

Younger participants had access to new opportunities to make alternative lifestyle choices which they learnt to implement whilst navigating traditional cultural expectations of what is deemed appropriate. They also considered the impact of said choices on their health and well-being. This includes increased awareness of their sexual, reproductive, and mental health:

“I think, maybe, if you’re not just talking about physical health, if you’re talking about mental health, in the Pakistani community, mental health isn’t acknowledged, it’s not something that’s ever talked about. If you’re depressed, you’d never go to a doctor, or get anti-depressants or anything like that, because it’s a problem that doesn’t exist”.

(18–29 years, second-generation, professional, IMD 1, area D)

Adopting a Western stance can at times empower participants to justify their choices, compared to the traditional Pakistani perspectives they had outlined, although this varied depending on what region of Pakistan families travelled from:

“‘Our nose would be cut off (losing honour) in the community, the *baradari*-ism (fraternity) that they will be from a different caste, that they are *Chaudhry*, that they are *Rajput*, that they are whatever’, so ‘if she marries into a different *baradari*, then our nose will be cut off’, that’s the biggest thing that affects our community, it’s like rust, it is eating away at us, that caste, marrying outside of the caste, that ‘she will cut our nose off in the *baradari’”*

(18–29 years, first-generation, manual worker, IMD 1, area F)

The differences and nuances within the Pakistani community means some families value traditional health and social practices more than others [10]. Participants acknowledged the cultural differences in attitudes towards educated women amongst Mirpuri and Azad Kashmiris, in contrast to those from cosmopolitan cities such as Islamabad or Lahore. Specific communities within the Pakistani diaspora ostracised women for being empowered and independent [6].

“I think it depends what part of Pakistan you come from, a lot. So, a lot of the people surrounding me they come from Mirpur, part of Pakistan where they’re quite strict, they don’t like their ladies to be working”.

(18–29 years, first-generation, professional, IMD 1, area E)

Participants bonded over shared experiences as migrants, Muslims, and women in England, navigating fluid identities and assimilation. In high ethnic-density areas of the West Midlands, pressure to conform to community norms- often set by elders and men- made balancing cultural and Western practices difficult [33]. While some found comfort in these communities, others faced challenges:

‘I think it depends where they are. Because if you go like, (area with a high Pakistani population), they’re very, just, about their bubble there and they’re not getting that much, you know, interaction with other people and stuff, or even other families, especially families which have marriages within people they know (consanguinity). And I think once that happens then they don’t get an opportunity to change, or like, see what’s going on in the world around them. So yeah, I think it’s about where you are and who you’re with”.

(18–29 years, first-generation, student, IMD 1, area E)

Living in culturally defined spaces created a dichotomy between orthodox and secular practices. This influenced how community members interact with each other and the type of social connections they formed. The following participant described the difficulty with social interactions involving religious community members who can be very critical of other people’s behaviour.

“So, we’ve got one part of the community, that is starting to value going out, how you look, materialistic stuff, and then you’ve got the other part that’s becoming isolated and insular from the rest of the community because it’s really gone into the religion. So, you’ve that two parts of the community. And the one part of the community which has become very orthodox it’s even more difficult to talk to them because then you’ve got these ‘oh, you can’t do this, you can’t do that’’.

(50 + years, first-generation, professional, IMD 7, area C)

#### The force and influence of the patriarchy

Misogyny and patriarchy in the Pakistani community manifest through control over women’s mobility, decision-making, domestic roles, appearance, and marriage choices. Women are expected to follow guidance from male family members, with unmarried women’s behaviour particularly monitored both inside and outside the home:

“I think to be honest; they’ve just learnt to deal with when I was very young my dad was, he was trying to get me into a scarf, he was trying to get me to wear the Asian dress of *shalwar kameez* and things like that, but it wasn’t working, it wasn’t happening.”

(18–29 years, second-generation, student, IMD 5, area M)

For first-generation participants this also transferred to their husbands once married. Such negotiations are challenges presented daily on how to practice lifestyle choices, where going against the values and beliefs of the community, set by men, can lead to tensions. Men act as gatekeepers and provide approval for the preservation of traditional practices and approval of novel lifestyle choices. However, attitudes are changing and where such beliefs exist addressing them remained challenging for participants especially when exercising lifestyle choices in the public domain in areas of high Pakistani density.

“Those girls have started running and they go to XX (park in centre of microcosm/recent migrant area), an Asian park like at least I still have respect for my culture and I won’t do it there, one, because my brothers would say ‘what the flip are you doing?’ and two, I just wouldn’t ‘cos I’d get in to trouble because it’s not socially accepted but those girls have actually started running this year”

(18–29 years, second-generation, professional, IMD 1, area F)

## Discussion

While CVD prevention was the initial rationale for exploring lifestyle behaviours, we found there are wider benefits, such as through diet and physical activity, that are shaped more by gender norms and identity expectations; hence a need that should be considered by policymakers in the framing of public health messages. This study has identified four areas within which Pakistani female participants faced challenges: (1) the ability to engage with the opportunities provided by work and education whilst abiding by traditional and cultural values; (2)adhering to social pressure on physical appearance; (3) the desire to find support that is free of judgement for pursuing alternative (to Pakistani culture) lifestyle choices, and (4) battling with the firmly embedded patriarchal views regarding women’s behaviour.

Our findings illuminate a cultural dichotomy that Pakistani women encounter; that is expectations regarding appearance and societal expectations remain high even once they are married and/or when older. The shift is visible for young women who are expected to have a modest yet attractive appearance to find a suitable spouse, whereas for older women or married women, modesty is prioritised. These expectations are rooted in traditional gender and community norms which emphasises modesty, honour and family reputation. Given their growing presence in the labour market, women have the means and opportunities to invest in their appearance more which challenges traditional first-generation perspectives of women’s roles, thus no longer solely focused on child rearing and caring for their spouse. Societal beauty standards still exist, there is generally more individual autonomy regarding appearance, with less direct pressure from family or community on maintaining modesty or aligning beauty with family reputation in the wider community.

Women in this study noted frequent physical comparison with others, often influenced by Western beauty ideals, leading to internalised slim body norms [40] and feelings of subordination [14, 36]. In Canada, South Asian women associated ‘looking good’ with social acceptance, while health remained a personal matter [14]. Modern self-care trends challenge traditional roles prioritising caregiving over personal well-being. Pakistani women navigate these shifts while balancing cultural norms, modesty, and Western ideals in both beauty and sports [2, 36], reflecting ongoing identity negotiations.

In the nineteenth century Edwin Long painted the *Babylonian wife market,* a provocative image depicting the auctioning of women for marriage, where the women are ranked based on their appearance [17]. The painting was inspired by the women’s suffrage movement, and it sheds light on the continuing challenges faced by Pakistani women today. Participants in this research tried to challenge the patriarchal views in a safe way, that women should be able to prioritise their own health, and not solely for attracting a suitable husband or looking after their families. Women should have the opportunity to showcase their abilities in numerous other ways to their community. Participants in this research provided examples of how they tried to challenge patriarchy and achieve opportunities to become more empowered by taking greater autonomy of their physical bodies.

The values and expectations placed on women varied by generation. Notably, half (*n* = 11) of the participants in the study were first-generation, married women. First-generation and recent migrant women maintained an expectation to prioritise their family life, whereas second and third- generation women were engaged within sub-cultures (e.g. Pakistani British Muslims), where greater opportunities to learn about diet and health were available. The influence between generations was beyond dyadic, where sharing values, such as those related to appearance, were not limited to direct, two-way relationships (e.g. mother-daughter), but extended across broader social and familial networks involving multiple women from different generations and backgrounds. Cultural values may be transmitted in more complex, collective ways, encompassing shared experiences among various women within the community, including those with different migrant statuses trying to navigate their identities as British Pakistani Muslim women in England. This also highlights the enduring influence of traditional marital and gender norms on health behaviours, emphasising the need for culturally tailored interventions accounting for generational and marital dynamics on associated practices.

The findings of this research highlight the need for policy makers to recognise community diversity in the context of a varied spectrum of cultural values [4]. Healthcare promotion should integrate cultural and religious considerations to better engage minority ethnic groups [22]. Greater knowledge transfer and co-production with local communities is needed to engage in lifestyle development. However, time and resource constraints in healthcare systems make personalised care challenging. Thus, broader community engagement with stakeholders and policymakers is essential to promote health awareness and preventive lifestyle choices [9]. Some women described older family members, community spaces, or women-led groups as key sources of encouragement, offering culturally sensitive support that bridged tradition with contemporary health goals.

Participants highlighted the need to challenge the gendered discourse prevalent in their community. For example, in health spaces, how care should be delivered, how women should approach each other and communication styles for lifestyle behavioural services designed for migrant communities rarely consider the needs of second and third generation members e.g. women’s health (e.g. contraception) is usually only discussed regarding family planning and not pre-marital relations.

Ali and Gavino [1] commented on Pakistani society as a patriarchal system where some men hold the decision-making role as heads of the family and where women feel vulnerable without their guidance. In the present findings, Pakistani families maintained these patriarchal structures, where the husband or father, as head of the house, used their position to determine the appropriateness of social behaviours. Hence, there is a need to involve men and encourage them to learn about the importance of women having greater control in decision-making especially at the minimum concerning their own health choices.

### Strengths and limitations

As far as we are aware, our findings are first to explore in-depth; how Pakistani women in the UK navigate the intersection between social capital, education, employment, and health within the context of cultural and religious expectations. By focusing on this specific cohort, the findings address a gap in existing literature on minority-ethnic communities and their health behaviours.

The research examines generational shifts in values and expectations among Pakistani women, highlighting how second- and third-generation experiences differ from first-generation counterparts. An interpretive approach explores cultural and religious barriers to self-care and health, linking social capital to chronic disease prevention in deprived areas. Expanding social networks through education and employment can challenge traditional gender roles.

These findings provide a snapshot of Pakistani women in the West Midlands, offering insights applicable to other migrant groups. Using multimedia preserved participant anonymity while capturing nuanced data. Including third-generation women, often underrepresented, enhances understanding of evolving cultural identities [30].

A reflexive approach was taken to consider the influence of a female, Pakistani interviewer completing interviews with Pakistani women. The interviewer was aware of cultural etiquette and appropriateness (e.g. wearing modest clothing) and participants were willing to open-up around personal issues, understanding that the researcher was aware of gender-norms when conducting the interviews. The multi-disciplinary research team (consisting of men and women from backgrounds in Psychology, medical sociology, and clinical backgrounds and of South Asian, British, and European descent) contributed to the analysis and interpretation of the findings to minimise bias and encourage reflection on broader cultural themes.

The sample was skewed with a greater number of women with higher education qualifications and fewer older participants. As with many qualitative studies, self-selection bias may have influenced participation, and the regional focus on the West Midlands limits generalisability, nevertheless allows for transferability to other South Asian or migrant communities. However, the sample size of 22 participants is appropriate for an in-depth qualitative study and allowed for thematic saturation and diversity across generations, migration status, and socio-economic background [39].

## Conclusion

This study highlights the cultural challenges Pakistani women face in adopting healthier lifestyles, where prioritising personal well-being can be seen as defying traditional family-first values. Women are encouraged to focus on attractiveness for marriage rather than physical or mental health. However, participants recognised the benefits of diet, exercise, and socialising beyond their immediate community. Empowering women requires culturally sensitive health guidance, such as exercise spaces that allow modest dress, healthier versions of traditional foods, and framing health as a collective value that strengthens family and community roles. These findings are particularly relevant for public health authorities, commissioners of culturally tailored services, and local community leaders involved in co-designing interventions. It is essential that Pakistani women are engaged as active partners in shaping knowledge and driving meaningful change.

## Data Availability

The dataset generated and analysed during the current study are not publicly available due to concerns over anonymity and confidentiality, but some information is available from the corresponding author on reasonable request.

## Abbreviations

CVD: Cardiovascular disease
UK: United Kingdom
IMD: Index of Multiple Deprivation
NHS: National Health Service
